# Cardiac protection of wogonin in mice with pulmonary fibrosis by regulating Sirt1/ γ-H2AX pathway

**DOI:** 10.3389/fphar.2025.1551141

**Published:** 2025-04-14

**Authors:** Libo Wang, Fei Lin, Runran Miao, Tianhao Zhao, Yuan Liu, Lin Yang, Min Zhang

**Affiliations:** ^1^ Collaborative Innovation Center of Henan Province for Green Manufacturing of Fine Chemicals, Key Laboratory of Green Chemical Media and Reactions, Ministry of Education, School of Chemistry and Chemical Engineering, Henan Normal University, Xinxiang, China; ^2^ Department of Cardiology, Life Science Research Center, The First Affiliated Hospital of Xinxiang Medical University, Xinxiang, Henan, China; ^3^ King’s College London British Heart Foundation Centre of Research Excellence, School of Cardiovascular and Metabolic Medicine & Sciences, London, United Kingdom

**Keywords:** heart failure, pulmonary fibrosis, wogonin, bleomycin, Sirt1

## Abstract

**Background:**

Clinical evidence suggests that pulmonary fibrosis (PF) and heart failure (HF) often co-exist; however, the specific impact of PF on HF remains underexplored. This gap in understanding complicates the management and treatment of HF in patients with PF.

**Objectives:**

To investigate the effects of PF on cardiac function and myocardial fibrosis using a mouse PF model and evaluate the therapeutic potential of wogonin, a flavonoid compound known for its anti-PF properties.

**Methods:**

A PF mouse model was established via intratracheal administration of bleomycin (BLM). Starting on day 8 post-BLM treatment, wogonin (50 mg/kg) was intraperitoneally administered every 2 days for 2 weeks. Cardiac function was assessed using echocardiography, while myocardial fibrosis was evaluated through Masson staining. *In vitro*, H9C2 cardiomyocytes were exposed to CoCl_2_ or H_2_O_2_ for 24 h with or without wogonin (20 μM) treatment. Apoptosis and DNA damage markers were analysed using immunofluorescence, immunoblotting, and the Comet assay. The interaction between wogonin and Sirt1 was examined using biotin-affinity pulldown assays and molecular docking simulations.

**Results:**

Mice with PF exhibited significant cardiac dysfunction and myocardial fibrosis. Wogonin treatment markedly improved ejection fraction and attenuated myocardial fibrosis in PF mice. Mechanistic studies revealed that wogonin alleviated DNA damage and cardiomyocyte apoptosis by upregulating Sirt1 and downregulating γ-H2AX expression. Docking simulations predicted that wogonin forms a stable complex with Sirt1 through hydrogen-bonding and hydrophobic interactions, which was further validated by biotin-affinity pulldown assays.

**Conclusion:**

Wogonin exerts protective effects against cardiac dysfunction and fibrosis in PF mice by modulating Sirt1/γ-H2AX-mediated pathways to reduce DNA damage and apoptosis. These findings suggest the potential of wogonin as a therapeutic agent for managing HF associated with PF.

## 1 Introduction

Idiopathic pulmonary fibrosis (IPF) is a chronic, progressive disease characterized by irreversible lung scarring and severely impaired lung function, with a median survival of approximately 4.5 years after diagnosis (Kistler et al., 2014). Clinical studies have highlighted cardiovascular disease (CVD) as the most prevalent comorbidity in IPF and a major risk factor for increased mortality (Kreuter et al., 2016). Cardiovascular manifestations associated with IPF include pulmonary hypertension (PH), coronary artery disease, cardiac remodeling, and ultimately heart failure (HF). Hypoxia and oxidative stress following pulmonary fibrosis (PF) further exacerbate these conditions (Almani et al., 2021). For the treatment of IPF-induced HF, it is essential to address both the underlying cardiac pathology and the pulmonary fibrosis. (Mosher and Mentz, 2020). Despite the significant clinical burden, the impact of PF on HF development and the underlying mechanisms remains poorly understood, posing challenges in managing HF in patients with PF.

HF is a debilitating condition affecting more than 64 million people worldwide (Savarese et al., 2023). Myocardial fibrosis is recognized as a critical component of adverse cardiac remodeling, driving structural and mechanical changes in the heart. These changes include cardiomyocyte apoptosis, hypertrophy, myocardial stiffness, chamber dilation, and ultimately cardiac dysfunction (Frangogiannis, 2019; Webber et al., 2020). The severity of myocardial fibrosis correlates with increased mortality in HF patients, yet the fundamental mechanisms underlying its progression remain insufficiently understood, hindering the development of effective therapies to reduce or reverse fibrosis.

Emerging evidence suggests that DNA damage and cardiomyocyte apoptosis play pivotal roles in the development of myocardial fibrosis (Chen et al., 2019; Rouhi et al., 2020). Among the implicated pathways, Sirt1, an NAD-dependent histone deacetylase, has been identified as a key mediator of DNA repair in cardiomyocytes. Sirt1 regulates the deacetylation and phosphorylation of H2AX, a crucial process for maintaining genomic stability (Kuno et al., 2023). Deficiency in Sirt1 has been linked to elevated levels of DNA damage, underscoring its protective role (Dobbin et al., 2013). However, the involvement of Sirt1 in cardiac fibrosis, particularly in the context of PF, and its potential as a therapeutic target, remain largely unexplored.

Wogonin (Wog), a flavonoid compound derived from the traditional Chinese herb *Scutellaria baicalensis*, exhibits diverse pharmacological properties, including anti-inflammatory (Ge et al., 2021), antioxidant (Lu et al., 2023), anti-fibrotic (Wang et al., 2024), anticoagulant (Wu et al., 2019), anticarcinogenic (Yang et al., 2020), and antiaging effects (Banik et al., 2022). Wogonin has demonstrated protective benefits against various cardiovascular diseases, including myocardial infarction (Bei et al., 2020), PH (Li et al., 2023) and HF (Khan et al., 2016). Additionally, our recent research showed that wogonin significantly attenuates lung fibrosis by reducing cellular senescence in a bleomycin-induced mouse model of PF (Wang et al., 2024). These findings raise an intriguing question: could wogonin provide cardioprotective effects in the setting of PF?

In this study, utilizing an established mouse model of PF, we found that mice with PF exhibited significant cardiac dysfunction and myocardial fibrosis. Importantly, treatment with wogonin markedly mitigated these pathological changes. Mechanistically, wogonin exerted its protective effects by modulating Sirt1/γ-H2AX-mediated pathways, thereby reducing DNA damage and cardiomyocyte apoptosis. These findings offer preliminary evidence supporting the potential clinical application of wogonin in managing HF associated with PF. Further studies are warranted to explore its therapeutic potential and underlying mechanisms in greater depth.

## 2 Materials and methods

### 2.1 Experimental mouse model of lung fibrosis

Male C57BL/6J mice (8–10 weeks) were purchased from Vital River Laboratory Animal Technology (Beijing, China). Mice were maintained under 12-h light and 12-h dark cycle from ZT0 to ZT12 at a temperature of 24 C ± 1°C and humidity of 50% ± 10%, free with water and diet. Mouse pulmonary fibrosis was induced by a single intratracheal instillation of 1.5 U/kg BLM (Macklin, Shanghai, China) as previous describe (Wang et al., 2024). Briefly, male 9- to 12-week-old C57BL/6 mice were anesthetized with 1% isoflurane and then intratracheally administered BLM in 50 μL of sterile saline. Control mice were administered the same volume of sterile saline. To test the therapeutic efficacy of wogonin in BLM-induced established fibrosis, wogonin (SW8020, Solarbio, Beijing, China) was intraperitoneally administered at 50 mg/kg/2-day starting at the day 8 after BLM treatment for 2 weeks, and the mice were sacrificed on day 21. Control mice received an equal volume of normal saline only. All animal care and experiments were conducted in compliance with the requirements of the National Act on the Use of Experimental Animals (China) and were approved by the Ethics Committee of the First Affiliated Hospital of Xinxiang Medical University (No. EC-022-129).

### 2.2 Echocardiography

Mouse cardiac echocardiography was performed as previously reported (Hancock et al., 2018). Standard 2D Echocardiography was performed under 1.5% isoflurane anesthesia at heart rates >400 beats/min, using a Vevo3100 ultrasound system with a 40 MHz linear probe (Visualsonics, Canada). Cardiac function was evaluated from parasternal short-axis images at the level of the papillary muscles and images were acquired using M-mode at a depth setting of 11 mm. Echocardiographic imaging and analyses were performed by an experienced echocardiographer who was blind to the animal groups.

### 2.3 Histology and immunohistochemistry

The mice were humanely euthanized, and tissue samples were rapidly harvested and washed in PBS to remove blood, fixed in 4% paraformaldehyde immediately, dehydrated, embedded in paraffin wax and sectioned (4 μm) for further use. Collagen deposition was stained using Masson’s trichrome kit (G1346, Solarbio, Beijing, China) and Sirius Red stain Kit (G1472, Solarbio, Beijing, China) according to the manufacturer’s instructions. Tissue sections for immunostaining were deparaffinized, rehydrated, and treated with citrate buffer to retrieve antigens. Subsequently, sections were incubated with α-SMA antibody (1:200 diluted, Huabio, Hangzhou, China), Sirt1 (1:400 diluted, Proteintech, Wuhan, China) and γ-H2AX antibody (1:300 diluted, Abcam, Cambridge, UK) at 4 °C overnight, following the recommendations of the manufacturers and protocols previously described (Liu et al., 2020). The sections were counterstained with hematoxylin and the positive fractional areas were quantified using ImageJ software (V1.53, National Institutes of Health, NIH).

### 2.4 TUNEL staining

Paraffin-embedded sections of the hearts were fixed in 4% paraformaldehyde and dewaxed. Terminal deoxynucleotidyl transferase dUTP nick end labeling (TUNEL) staining was performed in heart sections by using the One Step TUNEL Apoptosis Assay Kit (C1086, Beyotime Biotech, Beijing, China) in accordance with the manufacturer’s protocols. Images were captured using a fluorescence microscope (Nikon Confocal microscope) and were analyzed using ImageJ software for quantification.

### 2.5 Cell culture and treatment

The H9C2 cardiomyocytes (ATCC; Catalog number: CRL-1446) were cultured in Dulbecco’s modified Eagle’s medium (DMEM) (CORNING, Cat 10-013-CV, USA), supplemented with 10% (v/v) fetal bovine serum (FBS) (Corning Cat 35- 010-CV, USA) and penicillin–streptomycin (Gibco, Cat 15-140-122, USA) at final concentrations of 100 U/mL and 100 μg/mL, respectively. Cells were cultured at 37°C in a humidified atmosphere containing 5% CO_2_. The cells were incubated with CoCl_2_ (200 µM) to stimulate cells under oxygen disruption (Zhang et al., 2010), or H_2_O_2_ (200 µM) to stimulate oxidative stress for 24 h as previously described (Eleftheriadou et al., 2017). For rescue experiments, H9C2 cells were treated with wogonin at a concentration of 20 µM (Wang et al., 2024), EX527 at a concentration of 10 µM (Tang et al., 2022), and resveratrol at a concentration of 30 µM (Kuno et al., 2023).

### 2.6 Immunofluorescence

H9C2 cells in 24-well plates were permeabilized with 0.25% TritonX-100 in PBS for 10 min. After blocking with 5% BSA for 1 h at room temperature, then incubated with γ-H2AX antibody (ab81299, Abcam) overnight at 4°C. After washing 5 times with PBS, cells were incubated with Alexa Fluor secondary antibodies (A0423, Beyotime, Beijing, China) (1:300 dilution) for 1 h at room temperature in the dark. Cell nuclei were counterstained with DAPI. Fluorescent images were obtained using a Zeiss fluorescence microscope.

### 2.7 Comet assay

DNA strand breaks were analyzed by single cell electrophoresis (neutral comet assay) using a Comet assay kit (C2041M, Beyotime, Beijing, China) according to the manufacturer’s protocol. Briefly, H9C2 cells stimulated with CoCl_2_ or H_2_O_2_ were treated with or without wogonin for 24 h. The cells were collected and lysed, and then submitted to electrophoresis in an agarose gel at 1 V/cm for 20 min. After nuclear staining with Gel-red Dye, fluorescence images were captured by using a Zeiss fluorescence microscope. The comet tail moment was measured by using ImageJ software with an OpenComet plugin (Gorenne et al., 2013; Kuno et al., 2023).

### 2.8 Wogonin-biotin affinity pulldown assay

Biotinylated-wogonin (Bio-Wog) was purchased from ChongqingYusi Pharmaceutical Technology (ChongQing, China). The biotin-affinity pulldown assay was performed as previously described (Du et al., 2020). Briefly, Bio-Wog were synthesized and verified by mass spectrometry (data not shown). H9C2 cells were lysed in lysis buffer (50 mM Tris, pH 7.4, 150 mM NaCl, 0.1% (vol/vol) Triton X-100, 5 mM EDTA and protease inhibitors) and centrifuged at 12,000×g for 10 min at 4°C. Cell lysates were incubated with Bio-Wog (10 µM) or Bio for 2 h at room temperature. The supernatant was incubated with Streptavidin Magnetic Beads (Beyotime Biotech, China) for 4 h with gentle rocking. The beads were washed three times and then incubated in lysis buffer with 10 µM wogonin for 30 min to compete with Bio-Wog. Biotin alone was used as a control. Total lysates were used as an input control. The supernatant was boiled with 5x loading buffer, and the samples were loaded on a 10% polyacrylamide gel for Western blot analysis.

### 2.9 Western blotting

Total tissue or cell lysates were obtained in Cell Complete Lysis Buffer (P0037, Beyotime, Beijing, China). Equal amounts of protein were loaded in each lane of an SDS 8%–15% polyacrylamide gel and then transferred to polyvinylidene fluoride (PVDF) membranes. Western blot assays were performed by the standard method using the following primary antibodies: Sirt1 (13161-1-AP, Proteintech); γ-H2AX (ab81299, Abcam); Anti-Active Caspase 3 (bsm-33199m, Bioss); GAPDH (10494-1-AP, Proteintech); α-SMA (ET1607-53, Huabio). Protein bands were visualized using an enhanced chemiluminescence detection system on Amersham Imager 600 (GE Healthcare). The uncut gels were available in supplement. The protein levels were analyzed using ImageJ software (NIH, USA).

### 2.10 Serum biochemical assay

Plasma levels of alanine transaminase (ALT), aspartate transaminase (AST), blood urea nitrogen (BUN) and creatine kinase (CK) in mice were detected respectively by the commercial kit from Nanjing Jiancheng Co., Ltd. (Nanjing, China), according to the manufacturer’s instructions. TGF-β was measured by ELISA kit from Servicebio (Wuhan, China).

### 2.11 Molecular docking

AutoDock Vina 1.2.3 software was used to predict the potential molecular docking. The structure file of Sirt1 protein was derived from the RCSB protein crystal structure database (PDB ID: 4ZZH), and the structure of wogonin was constructed and optimized by Chem3D software. The structure files of wogonin and Sirt1 protein were operated by AutoDockTools software to add H atoms, add Gasteiger-Hücker empirical charges, combine non-polar hydrogen, and set rotatable bonds, among which the σ bonds between heavy atoms in the structure of wogonin were set as rotatable bonds, and the Sirt1 protein was regarded as a rigid structure. After that, a 50 × 50 × 50 Å docking square box was set at the cavity site of the Sirt1 protein by the AutoDock Vina program, and to perform conformational search and energy optimization in the square box for 200 docking times independently. Finally, the optimal binding conformation of wogonin-Sirt1 protein was selected from the docking results (absolute largest value of the score), the intermolecular interaction was visualized by using PyMOL software (version 2.2, https://PyMOL.org/2/).

### 2.12 Statistics

All data in this study were presented as mean ± SEM. Comparisons of groups were performed using one or two-way ANOVA, as appropriate. A post-hoc Tukey’s test was performed to isolate differences. *p*-values <0.05 were considered statistically significant. GraphPad Prism 9.0 statistical software was used for all the data analyses involved in this study.

## 3 Results

### 3.1 Wogonin alleviates BLM-induced pulmonary fibrosis in mice

**FIGURE 1:**
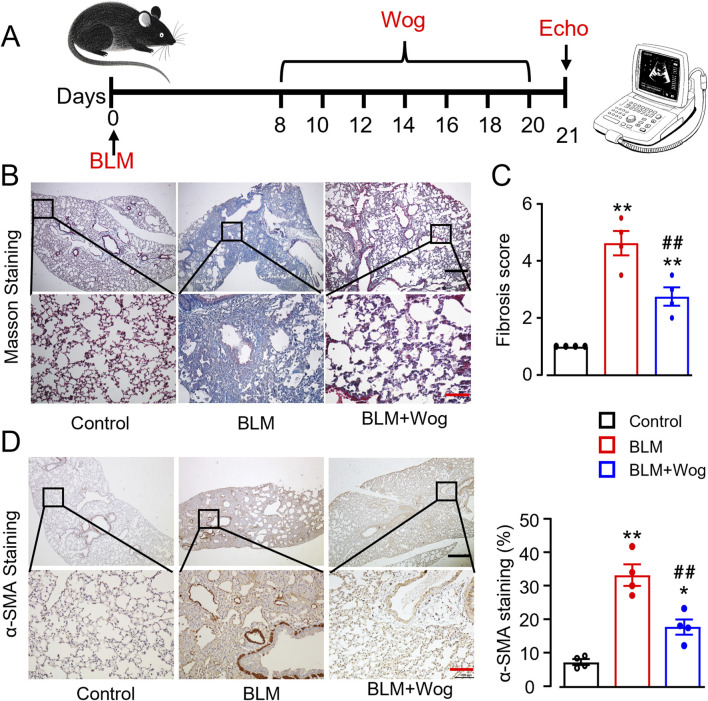
Wogonin alleviates bleomycin-induced mouse pulmonary fibrosis. **(A)** Timeline of wogonin administration. **(B)** Representative images of Masson staining with the lung sections, and **(C)** Fibrosis scores based on stained lung sections. N = 4 mice/group. **(D)** Immunohistochemical analysis of α-SMA with the lung tissues, and relative quantification of α-SMA at the right. Scale bars, 500 µm (black bars), 100 µm (red bars). N = 4/group, *p < 0.05, **p < 0.01, compared with control animals; ##*p* < 0.01, compared with BLM group, one-way ANOVA with a post-hoc Tukey’s test. All data are presented as the mean ± SEM. BLM, bleomycin; Wog, wogonin.

A pulmonary fibrosis (PF) mouse model was successfully established via intratracheal administration of bleomycin (BLM, 1.5 U/kg), as previously described (Wang et al., 2024). Wogonin (50 mg/kg) was administered intraperitoneally every 2 days for 2 weeks, starting on day 8 after BLM treatment (Figure 1A). With safety concern regarding the *in vivo* use of wogonin, we first examined if wogonin had any side effects in mice. There was no evidence of liver damage or renal dysfunction with wogonin intraperitoneal administration after BLM treatment by histology and plasma biochemical analysis (Supplementary Figures S1A, B). Interestingly, the levels of serum creatine kinase (CK) were obviously increased in PF mice, indicating the myocardium damage in PF mice (Supplementary Figure S1B). Importantly, collagen deposition, evaluated using Masson’s trichrome staining, showed that BLM induced significant PF, which was markedly reduced by wogonin treatment (Figures 1B, C). Immunohistochemical analysis revealed that wogonin significantly decreased the expression of α-smooth muscle actin (α-SMA), a marker of fibrosis, in the lungs of BLM-treated mice (Figure 1D). These results confirmed the successful establishment of the PF model and demonstrated that wogonin effectively attenuates BLM-induced PF in mice.

### 3.2 Wogonin protects against cardiac dysfunction in mice with pulmonary fibrosis

**FIGURE 2:**
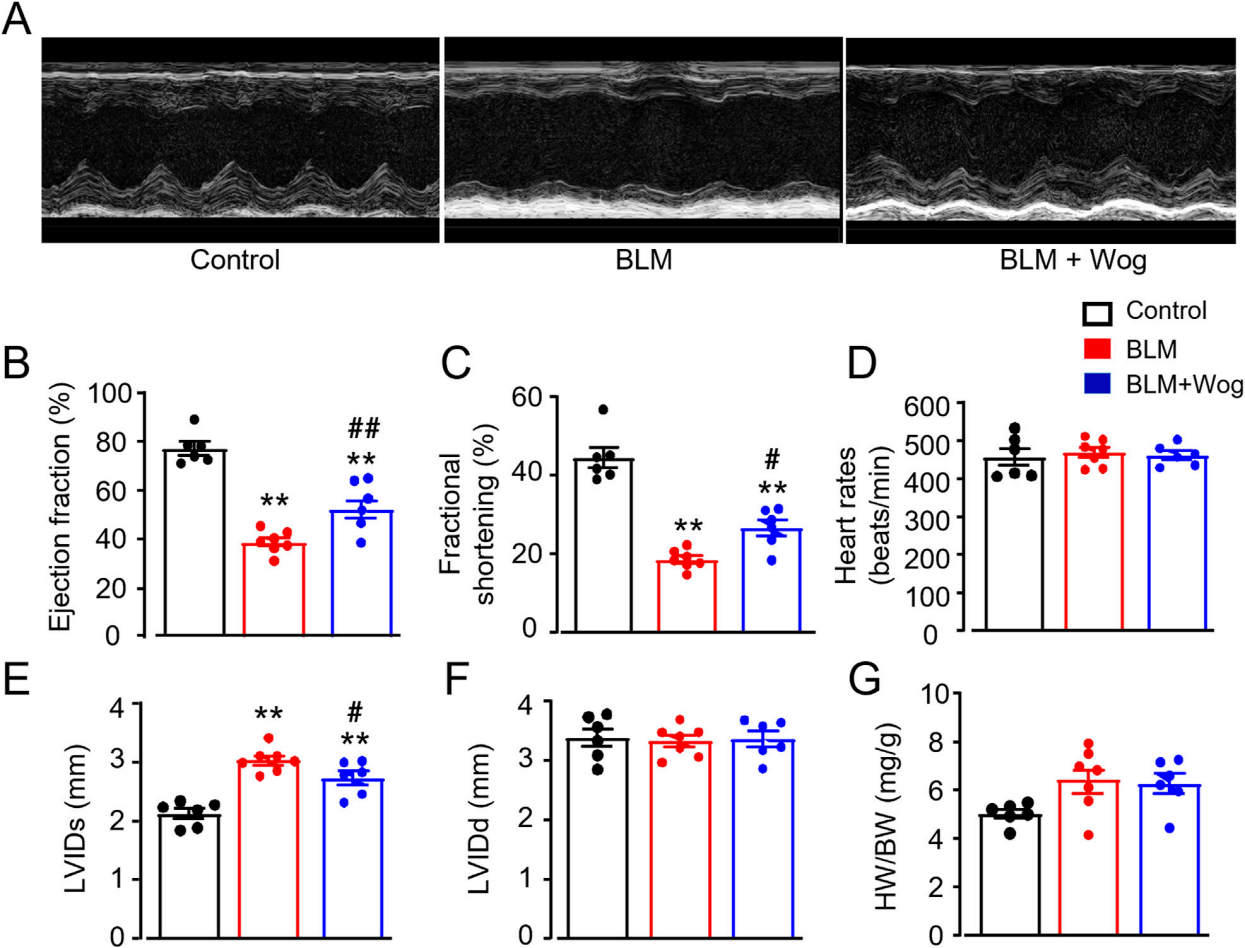
Wogonin protects against cardiac dysfunction in mice with pulmonary fibrosis. **(A)** Representative M-mode echocardiographic imagings. **(B)** Ejection fraction; **(C)** Fractional shortening; **(D)** Heart rates; **(E, F)** Left ventricle internal diameter at end-systole (LVIDs) and at end-diastole (LVIDd); **(G)** Heart weight-to-body weight ratio (HW/BW). N = 6-7 mice/group. **p < 0.01, compared with control animals; #*p* < 0.05, ##*p* < 0.01, compared with BLM group, one-way ANOVA with a *post hoc* Tukey’s test. All data are mean ± SEM. BLM, bleomycin; Wog, wogonin.

We next examined whether PF mice develop cardiac abnormalities and whether wogonin provides cardiac protective effects. Cardiac function was assessed using two-dimensional echocardiography (Figure 2A). PF mice exhibited impaired cardiac function 3 weeks after BLM treatment, with significantly reduced ejection fraction (EF) and fractional shortening (FS) compared to controls (Figures 2B, C). Notably, wogonin-treated PF mice showed improved cardiac function, with better-preserved EF and FS (Figures 2B, C). There were similar heart rates among groups (Figure 2D). Furthermore, mice in BLM groups also exhibited an increased left ventricle internal diameter at end-systole (LVIDs) compared to controls, which was significantly attenuated after wogonin treatment (Figure 2E). Interestingly, there were no alterations of left ventricle internal diameter at end-diastole (LVIDd) (Figure 2F), indicating no left camber dilatation. BLM-treated mice appeared to have enlarged hearts, but the heart weight to body weight ratios (HW/BW) did not reach statistical significance. HW/BW were also similar after wogonin treatment compared to PF group (Figure 2G). These results suggest that wogonin attenuates the progress of cardiac dysfunction in mice with pulmonary fibrosis, without the effect on cardiac hypertrophy.

### 3.3 Wogonin suppresses cardiac fibrosis

**FIGURE 3:**
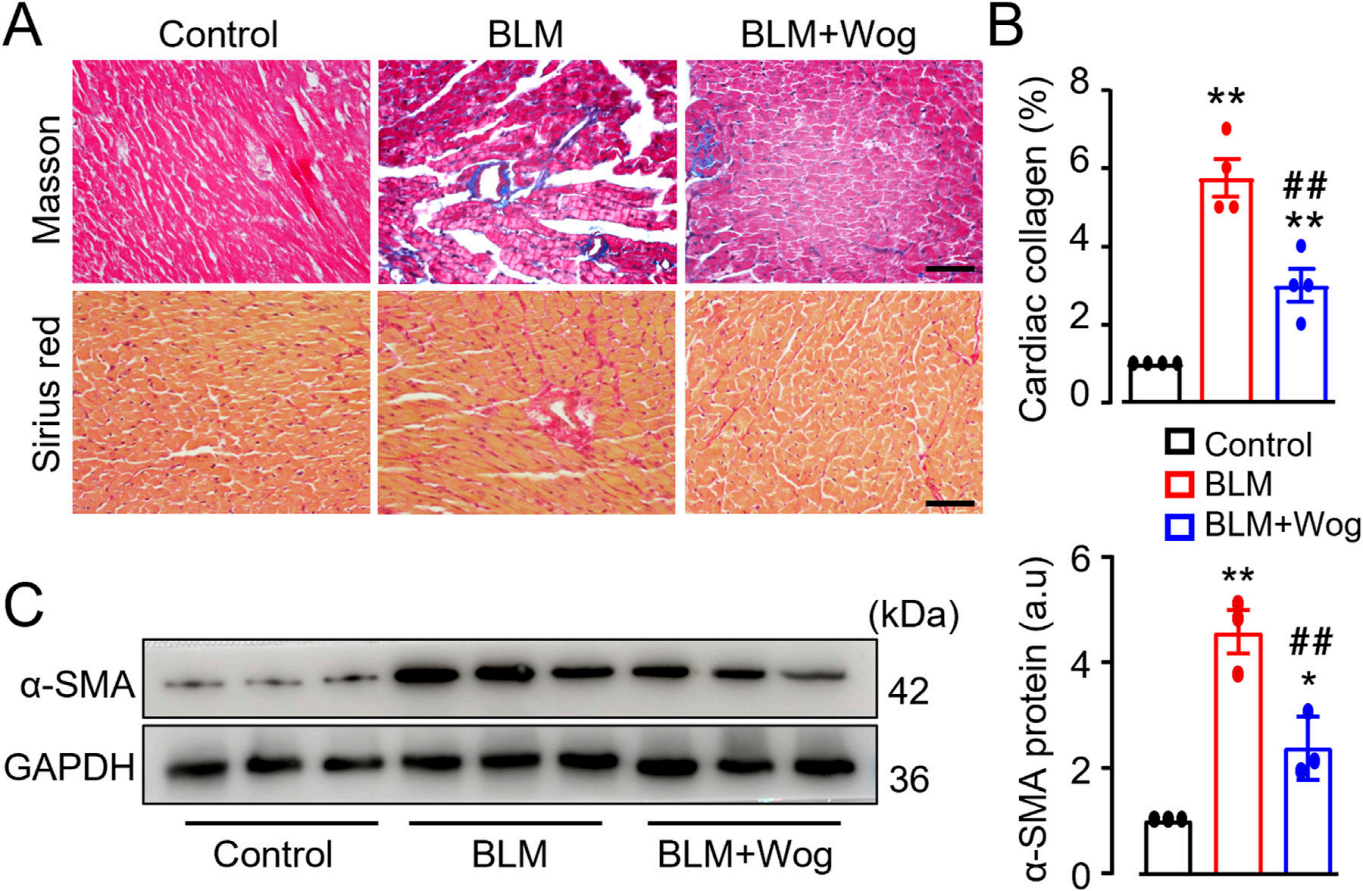
Wogonin suppresses cardiac fibrosis. **(A)** Representative images with Masson staining and Sirius red staining. Scale bars, 50 µm. **(B)** Relative quantification of collagen deposition assayed by Masson staining, n = 4/group; **(C)** The protein levels of α-SMA by Western blots. Mean data at the right, GAPDH as an internal control; n = 3/group; *p < 0.05, **p < 0.01 compared with controls; ##*p* < 0.01, compared with BLM, one-way ANOVA with a post-hoc Tukey’s test. All data are presented as the mean ± SEM. BLM, bleomycin; Wog, wogonin.

To further evaluate the effect of wogonin on structural changes of the hearts in mice with PF, Sirius red staining and Masson staining were used to examine myocardial fibrosis. PF mice showed significantly aggravated cardiac fibrosis compared to controls, as evidenced by increased collagen deposition (Figures 3A, B). Immunoblot analysis further confirmed elevated levels of α-SMA, a marker of myocardial fibrosis, in PF mouse hearts (Figure 3C). Strikingly, wogonin treatment significantly reduced cardiac fibrosis and α-SMA expression (Figures 3A–C). TGF-β is a well-established cytokine that drives tissue fibrosis. Interestingly, serum levels of TGF-β were elevated in PF mice but were significantly reduced following wogonin treatment (Supplementary Figures S2). These findings suggest that wogonin exerts a beneficial effect on cardiac function, likely by inhibiting systemic inflammation and myocardial fibrosis in PF mice.

### 3.4 Wogonin mitigates myocardial DNA damage and apoptosis

**FIGURE 4:**
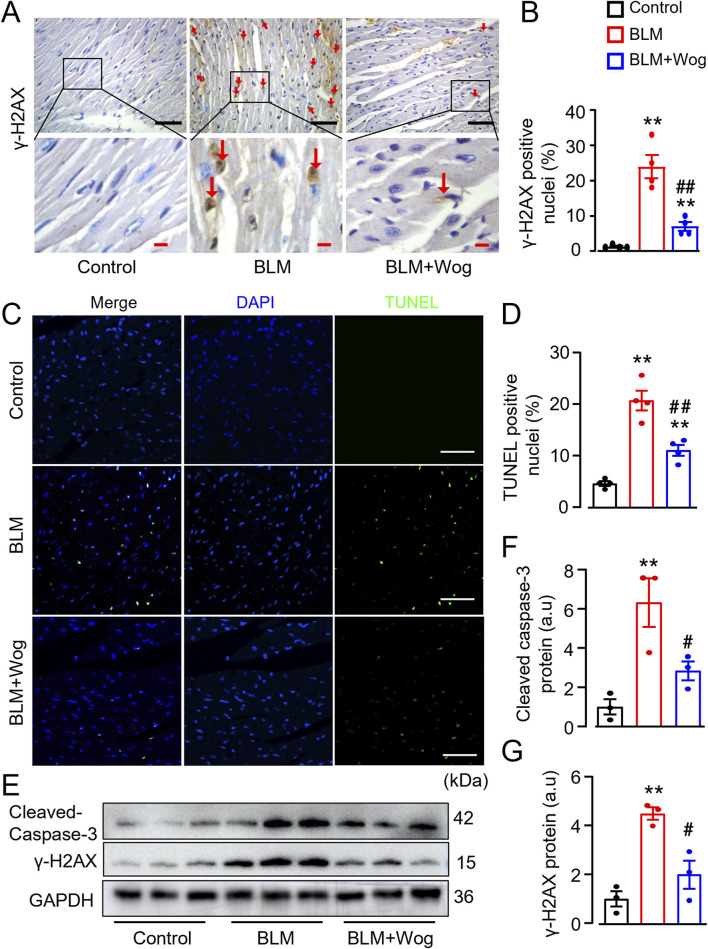
Wogonin mitigates myocardial DNA damage and apoptosis. **(A)** Immunohistochemical analysis of γ-H2AX in the hearts. Scale bars, 50 µm (black bars), 10 µm (red bars); **(B)** Relative quantification of γ-H2AX staining; **(C)** Apoptosis evaluated by TUNEL staining in heart sections. Scale bar: 100 μm; **(D)** Quantitation of TUNEL positive staining; **(E)** Protein levels of cleaved-caspase 3 and γ-H2AX in heart tissues by Westerns; **(F, G)** Mean data of protein immunoblots; n = 3–4/group; **p < 0.01 compared with controls; #*p* < 0.05, ##*p* < 0.01 compared with BLM groups, one-way ANOVA with a post-hoc Tukey’s test. All data are presented as the mean ± SEM. BLM, bleomycin; Wog, wogonin.

DNA damage and cardiomyocyte apoptosis are key contributors to cardiac fibrosis (Wu et al., 2022; Zhao et al., 2022). To explore the potential mechanisms underlying the beneficial roles of wogonin in the heart, we applied immunohistology and TUNEL assay to detect the expression of DNA damage marker γ-H2AX and apoptotic cells in the myocardium of PF mice with or without wogonin treatment. Immunostaining revealed the increased γ-H2AX expression mainly in the cardiomyocytes in the heart of PF mice, which was significantly diminished by wogonin treatment (Figures 4A, B). In line with this, the TUNEL positive cells in PF mouse hearts were markedly increased compared with control groups, treatment of wogonin significantly reduced apoptosis in the hearts (Figures 4C, D). Western blots also showed that the protein levels of γ-H2AX and apoptotic marker cleaved caspase-3 were significantly attenuated in the hearts of wogonin-treated group compared to PF mice (Figures 4E–G). These results suggest that the protective effect of wogonin against cardiac fibrosis is, at least partially, through the mitigation of DNA damage and apoptosis in the heart.

### 3.5 Wogonin attenuates DNA damage in H9C2 cardiomyocytes

**FIGURE 5:**
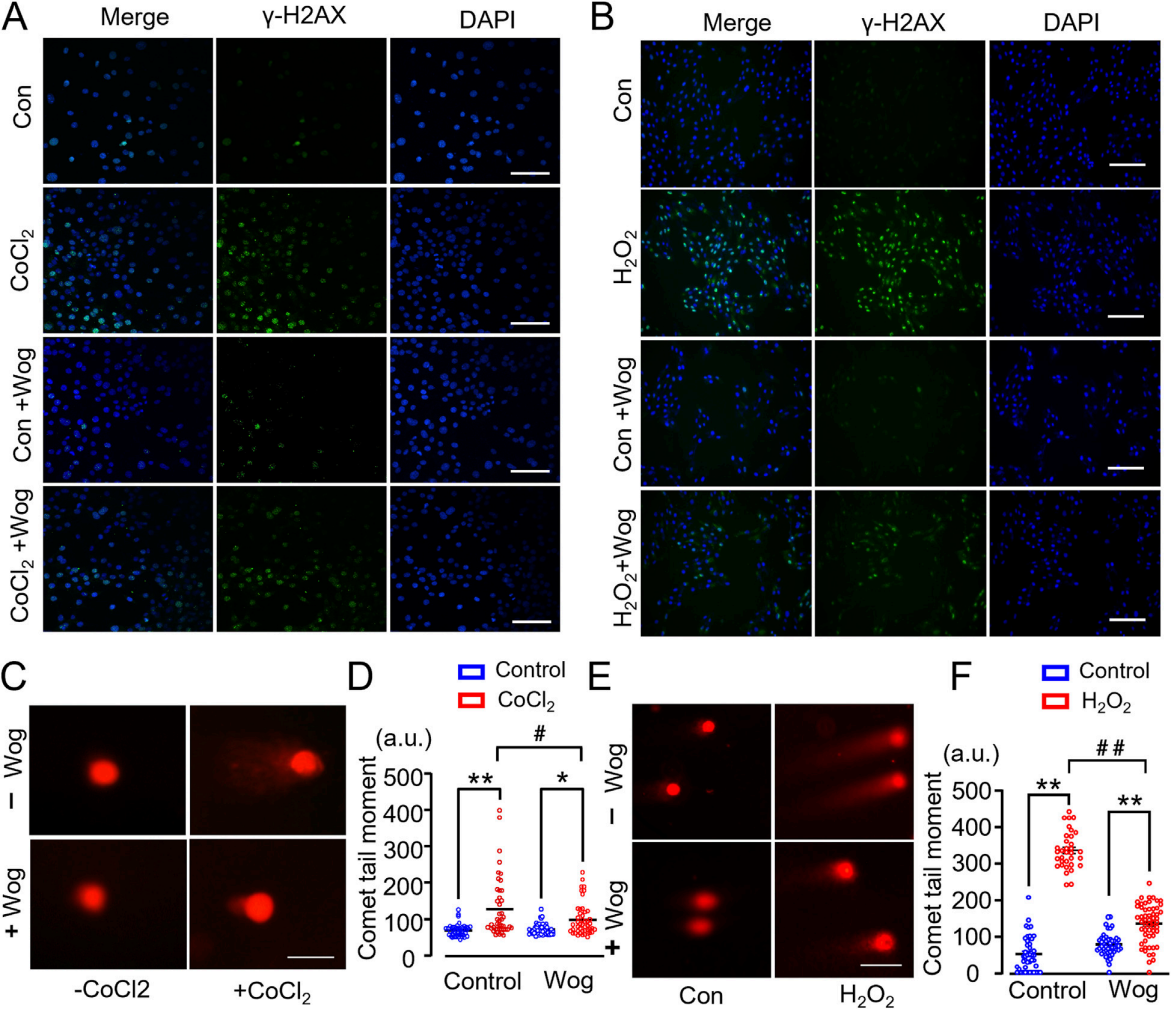
Wogonin attenuates DNA damage in H9C2 cardiomyocytes. **(A, B)** Immunofluorescence staining of γ-H2AX in H9C2 cells induced by CoCl_2_ and H_2_O_2_ respectively. Scale bar, 50μm; **(C, E)** Representative images of neutral comet assay in CoCl_2_ or H_2_O_2_-stimulated H9C2 cells with or without wogonin treatment. Scale bar, 50 μm. **(D, F)** Quantification of comet tail moment (a.u., arbitrary units) shown at the right respectively. At least 50 cells/group. *p < 0.05, **p < 0.01, compared with controls; #*p* < 0.05, ##*p* < 0.01, compared with respective CoCl_2_ or H_2_O_2_ group. 2-way ANOVA with a post-hoc Tukey’s test. All data are presented as the mean ± SEM.

Chronic hypoxia, which is a cardinal feature of PF due to the severe decline in pulmonary function, has detrimental impact on the heart including structural damage and cardiac dysfunction. To explore wogonin’s effects on DNA damage *in vitro*, we used CoCl_2_ to simulate hypoxia in cultured H9C2 cardiomyocytes. As expected, presence of CoCl_2_ increased the expression of DNA damage marker γ-H2AX by immunofluorescent staining (Figure 5A). This was further supported by a neutral Comet assay, which is another widely used method to monitor DNA damage *in vitro* (Martinet et al., 2002) (Figures 5C, D). Notably, 20 μM wogonin markedly diminished γ-H2AX levels and decreased the length of comet tail moment compared with that in H9C2 cells induced by CoCl_2_ alone (Figures 5A, C, D). Prolonged hypoxia can elicit oxidative stress which is a major player in cardiac fibrosis. H_2_O_2_ is a well-established inducer of DNA damage. Thus, we treated H9C2 cardiomyocytes with H_2_O_2_ at the concentration of 200 μM with or without wogonin, then examined DNA damage. Unsurprisingly, H_2_O_2_ induced clearly DNA damage in H9C2 as revealed by the increased expression of γ-H2AX and the length of comet tail moment; the effects were significantly attenuated by wogonin treatment (Figures 4B, E, F). These results demonstrate that wogonin protects against DNA damage induced by hypoxia and oxidative stress.

### 3.6 Wogonin diminishes DNA damage via upregulation of Sirt1

**FIGURE 6:**
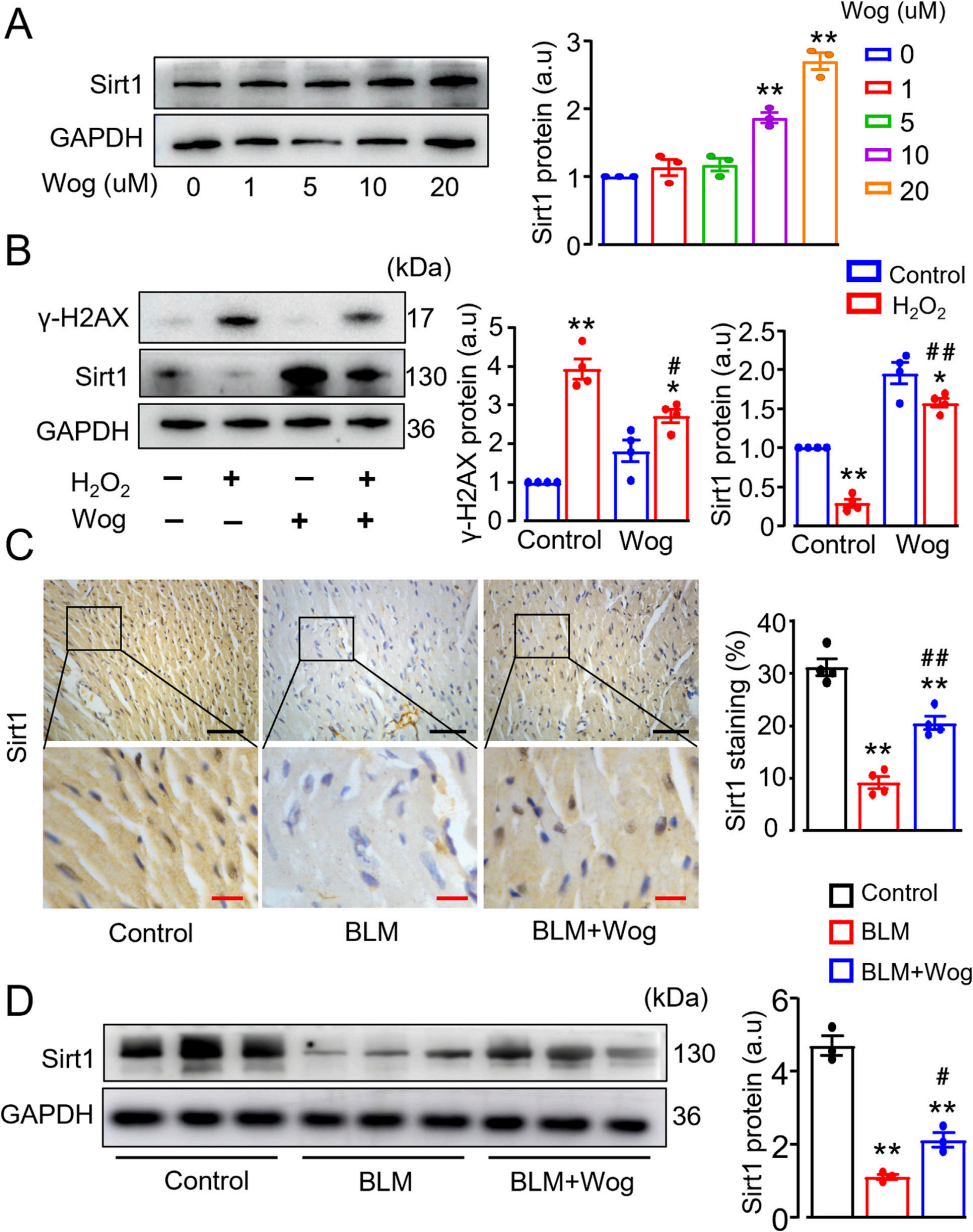
Wogonin diminishes DNA damage via upregulation of Sirt1. **(A)** Western blot analysis of Sirt1 proteins in H9C2 cells following the treatment with different concentrations of wogonin (0–20 μM) for 12 h. Mean data shown at the right. N = 3/group; **p < 0.01 compared to non-treatment; **(B)** Protein levels of γ-H2AX and Sirt1 in H_2_O_2_-stimulated H9C2 cells with or without wogonin treatment. Mean data at the right. N = 3/group; *p < 0.05, **p < 0.01, compared with controls; #*p* < 0.05, ##*p* < 0.01, compared with H_2_O_2_ group; **(C)** Immunohistochemical analysis of Sirt1 in the mouse heart sections. Scale bars, 50 µm (black bars), 10 µm (red bars). Relative quantification of Sirt1 shown at the right. N = 4/group; **(D)** Western blot analysis of Sirt1 proteins in mouse heart tissues. Mean data at the right, n = 3/group. **p < 0.01, compared with control animals; #*p* < 0.05, ##*p* < 0.01, compared with BLM group, one-way or two-way ANOVA with a post-hoc Tukey’s test. All data are presented as the mean ± SEM. BLM, bleomycin; Wog, wogonin.

Previous works showed that Sirt1-mediated γ-H2AX pathway is crucial regulator in DNA damage response (Gorenne et al., 2013; Bartoli-Leonard et al., 2020; Kuno et al., 2023). We found that treatment of H9C2 cells with wogonin for 12 h significantly increased Sirt1 protein levels in a dose-dependent manner, with maximal effects at 20 μM (Figure 6A). Stimulation of H_2_O_2_ significantly elevated γ-H2AX expression, along with reduced Sirt1 levels by immunoblots (Figure 6B). By contrast, treatment with wogonin significantly inhibited γ-H2AX expression, accompanied by the enhanced Sirt1 protein levels compared to H_2_O_2_ stimulation alone (Figure 6B), supporting the beneficial effect of wogonin against H_2_O_2_-induced DNA damage by modulating Sirt1/γ-H2AX pathway. To further investigate the role of Sirt1 in DNA damage, we tested the effects of the Sirt1-specific inhibitor EX527 and the well-known Sirt1 agonist resveratrol. As expected, EX527 completely abolished wogonin’s protective effects against H_2_O_2_-induced DNA damage in H9C2 cells, as indicated by the increased expression of γ-H2AX protein. By contrast, resveratrol significantly reduced γ-H2AX levels following H_2_O_2_ stimulation, mirroring the protective effects of wogonin (Supplementary Figures S3). These findings demonstrate the critical involvement of Sirt1 in wogonin’s protective mechanism against DNA damage. Moreover, the expression of Sirt1 was downregulated in the hearts of PF mice as revealed by both immunohistochemistry and immunoblots compared to control group, whereas wogonin treatment significantly increased the levels of Sirt1 (Figures 6C, D), highlighting the importance of Sirt1 in wogonin’s cardioprotective effects.

### 3.7 Sirt1 is a direct target of wogonin

**FIGURE 7:**
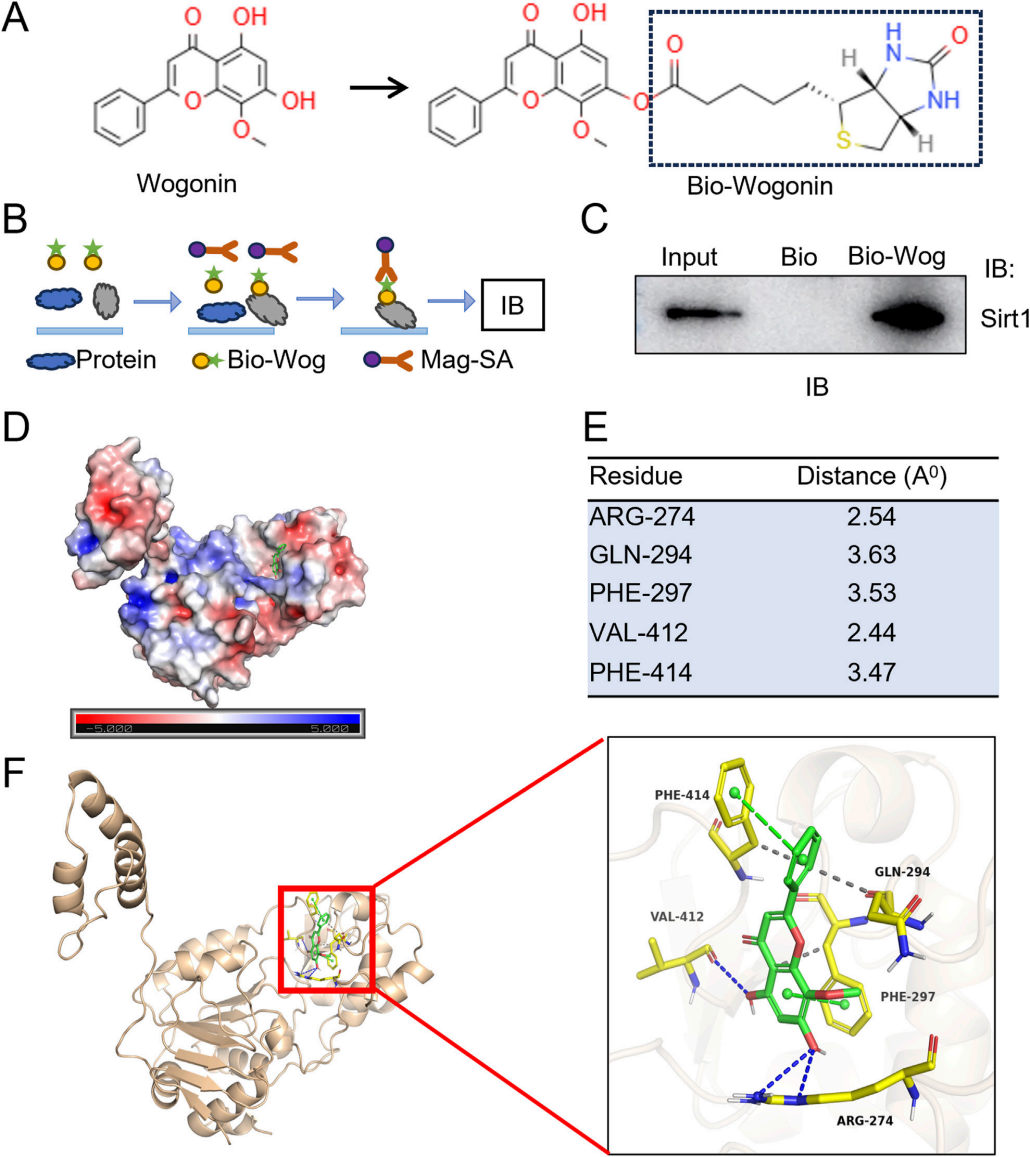
Sirt1 is a direct target of wogonin. **(A)** Chemical structure of wogonin and biotin-labeled wogonin (Bio-Wog); **(B)** Schematic showing steps for biotin-affinity pull-down assay; **(C)** Physical association of Sirt1 and wogonin evaluated by pull-down assay. Biotinylated wogonin (Bio-Wog) was used to pull-down Sirt1 with H9C2 lysates. Biotin alone as a control, total lysates as input; **(D)** Distribution of molecular electrostatic potential presented on Sirt1 (PDB code: 4ZZH); Blue and red color are the positive and negative potential, respectively. 3D molecular surface representation of the superimposition of wogonin (green sticks) against Sirt1; **(E)** The amino acid residues of Sirt1 with potential polar bonds with wogonin and the distance between each residue and wogonin; **(F)** 3D cartoon depiction of molecular interaction of docking complex between Sirt1 (yellow) and wogonin (green). The docking investigation revealed the presence of hydrogen bonds (blue dotted lines) and π-π stacking (green dotted lines).

To investigate the potential physical association between Sirt1 and wogonin, we synthesized a biotin-labeled wogonin (Bio-Wog) (Figure 7A) and performed biotin-affinity pull-down assays with H9C2 cell lysates (Figure 7B). Immunoblot revealed a direct interaction between wogonin and Sirt1 (Figure 7C). To explore the possible binding sites of wogonin with Sirt1 (PDB ID:4ZZH), the computational molecular docking analysis was conducted to predict the orientation of wogonin binding in the allosteric sites of Sirt1. Molecular electrostatic potential (MEP) was calculated to illustrate the molecule’s charge distributions and visualize the reactive sites interaction of Sirt1 and wogonin (Figure 7D). The modeling of docking simulation revealed that wogonin could interact with the amino acid residues on the catalytic sites of Sirt1 (Davenport et al., 2014) (Figure 7E), with the docking score (binding energy) of −8.060 kcal/mol. Furthermore, hydrophobic interactions were observed at residues GLN294, PHE297, and PHE414, while hydrogen bonds were formed at ARG274 and VAL412 (Figure 7F). These findings suggest that wogonin is a potent activator of Sirt1, directly associating with its active sites and stabilizing the protein.

## 4 Discussion

In the present study, we demonstrate that: 1) mice with PF develop cardiac dysfunction and myocardial fibrosis; 2) wogonin attenuates both lung fibrosis and cardiac fibrosis, effectively mitigating the progression of cardiac dysfunction; 3) the Sirt1/γ-H2AX pathway is critically involved in DNA damage and cardiomyocyte apoptosis, key contributors to cardiac fibrosis and dysfunction; 4) wogonin directly interacts with Sirt1, reducing DNA damage and apoptosis. These findings suggest that wogonin holds promise as a potential cardiac protective agent in the disease setting of PF.

CVD is among the most common comorbidities in patients with IPF (Mosher and Mentz, 2020; Sinha et al., 2024). The early symptoms of IPF, such as exertional dyspnea, cough, and reduced exercise tolerance, closely resemble those of HF (Mosher and Mentz, 2020). Notably, IPF patients are four times more likely to develop HF and exhibit higher risks of both all-cause and cardiovascular mortality (Koteci et al., 2022). Consistent with these clinical observations, our preclinical model showed that PF in mice leads to evident cardiac dysfunction. Despite the clinical significance of cardiovascular manifestations in PF, the mechanisms underlying the development of cardiac dysfunction in this context remain poorly understood.

Cardiac fibrosis, characterized by excessive collagen deposition in the myocardium, plays a central role in adverse cardiac remodeling. Evidence strongly suggests that myocardial fibrosis independently drives the development of cardiac dysfunction, ultimately leading to HF (Frangogiannis, 2021; López et al., 2021). In this study, PF-induced cardiac phenotypic changes in mice were marked by significant increases in myocardial fibrosis, as revealed by Sirius red and Masson staining and elevated levels of the fibrotic marker α-SMA. Interestingly, these changes occurred without cardiac hypertrophy, underscoring the pivotal role of fibrosis in HF progression associated with PF.

The mechanisms of myocardial fibrosis are multifaceted and involve persistent inflammation, hypoxia, cytokine storms, oxidative stress, and mitochondrial dysfunction (Ouyang et al., 2024) —all of which contribute to HF through the upregulation of myocardial fibrosis. These processes are closely linked to DNA damage and cardiomyocyte apoptosis (Li et al., 2021; Goerlich et al., 2024). Growing evidence suggests that DNA damage is a key driver of fibrotic remodeling in myocardial fibrosis and idiopathic pulmonary fibrosis (Zhao et al., 2022; Zhu et al., 2022; La Torre et al., 2023). Targeting DNA damage repair systems, such as poly (ADP-ribose) polymerase (PARP), is critical for the survival of damaged cells. PARP inhibitors, including 4-aminobenzamide and Olaparib, have been shown to protect against cardiac fibrosis induced by diabetic or doxorubicin-induced cardiomyopathy (Zakaria et al., 2016; Elkatary et al., 2023). DNA damage has also been shown to activate the transforming growth factor-β (TGF-β) and mitogen-activated protein kinase (MAPK) signaling pathways, which promote fibroblast activation and collagen synthesis, driving myocardial fibrosis (Lucas et al., 2015; Ko et al., 2022). Additionally, DNA damage induces cardiomyocyte apoptosis, leading to collagen deposition in the myocardium (Rath et al., 2024). Cardiomyocytes account for ∼80% of the cellular volume and 30%–40% of the total cell population in the heart. It is well reported that cardiomyocyte apoptosis plays the causal role in myocardial fibrosis and heart failure (Wencker et al., 2003; Henpita et al., 2023). In our study, we observed increased DNA damage and cardiomyocyte apoptosis in the hearts of PF mice, further implicating these mechanisms in PF-associated cardiac dysfunction. Of note, plasma TGF-β levels were elevated in PF mice, and treatment with wogonin significantly reduced the circulating levels of TGF-β. This suggests that, in addition to its direct protection in the heart, wogonin likely exerts a global beneficial effect by attenuating systemic hypoxia and inflammatory cytokine production through the cardiopulmonary axis.

Previous studies have showed that DNA damage could be mitigated by Sirt1 activation through mediating the deacetylation and phosphorylation of H2AX in cardiomyocytes (Bartoli-Leonard et al., 2020; Kuno et al., 2023). Sirt1 plays a crucial pathophysiological role in multiple biological processes including in the cardiovascular system, such as myocardial infarction, arrhythmia, heart failure, myocardial remodeling and myocardial fibrosis (Chen et al., 2023; Zhang et al., 2024). Sirt1 is important in mitigating DNA damage by regulating the DNA damage response (DDR). It achieves this by deacetylating and phosphorylating H2AX in cardiomyocytes, thereby maintaining genomic stability under stress (Kuno et al., 2023; Rasti et al., 2023). Sirt1 also promotes repair processes such as nucleotide excision repair (NER), homologous recombination, and non-homologous end joining (Ming et al., 2010; Zhang et al., 2016; Rasti et al., 2023). Our findings confirm the involvement of the Sirt1/γ-H2AX pathway in the myocardial fibrosis observed in PF mice. Activating Sirt1 appears to exert protective effects by stabilizing the genome and preventing fibrotic remodeling in the myocardium.

Wogonin has been reported to exert therapeutic effects on cardiovascular diseases such as atherosclerosis, coronary heart disease, myocardial infarction, pulmonary hypertension, and HF (Tan et al., 2022), indicating its broad clinical application potential (Huynh et al., 2020). Interestingly, the lungs and heart are two of the top four major organs capable of detecting accumulated wogonin, which confirms sufficient bioavailability for target engagement (Talbi et al., 2014). Our recent work demonstrated that wogonin significantly attenuates lung fibrosis (Wang et al., 2024), and previous studies have shown its efficacy in mitigating acute lung injury (Ge et al., 2021). However, its role in PF-associated cardiac dysfunction was previously unexplored.

In this study, we report for the first time that wogonin exerts cardiac protective effects in PF. Wogonin inhibited DNA damage and cardiomyocyte apoptosis, thereby reducing myocardial fibrosis and improving cardiac function. Mechanistic studies revealed that wogonin activates Sirt1 and downregulates γ-H2AX expression. Notably, docking simulations and biotin pulldown assays demonstrated a direct interaction between wogonin and Sirt1. Wogonin binds to the active sites of Sirt1 through hydrogen and hydrophobic bond interactions, stabilizing the protein and enhancing its activity. Sirt1 comprises distinct structural regions, including an NAD^+^-binding domain, a helical module, a Zn^2 +^-binding module, a pseudo-substrate peptide (T, tail), C-terminal regulatory segment (CTR) and a catalytic core. The catalytic core, comprising 277 residues, is divided into two subdomains: the helical module (residues 269–324) and the Zn2+-binding module (residues 362–419) (Davenport et al., 2014; Medoro et al., 2023). Wogonin binds to specific residues (ARG274, GLN294, PHE297, VAL412, and PHE414) within the Sirt1 catalytic pocket, influencing ligand stability and promoting Sirt1 activation.

There are some important limitations in this study. First, while BLM does not exhibit the same level of direct cardiotoxicity as agents like doxorubicin, it may still contribute to cardiovascular complications, particularly when used in combination with other chemotherapy drugs (Lauritsen et al., 2020). Moreover, the BLM-induced PF model differs from IPF, thus the direct effects of BLM on the heart require further investigation. Second, the *in vitro* study shows that the beneficial effect of wogonin against DNA damage in H9C2 cells can be abolished by Sirt 1 specific inhibitor EX-527, the intrinsic role of Sirt, however, should be validated using cardiomyocyte-specific gene-manipulated models with gain or loss of function. Third, the direct interaction between wogonin and Srit1 needs to be further determined by complementary biophysical methods e.g., cellular thermal shift assay (CETSA) or surface plasmon resonance (SPR) analysis. Fourth, wogonin has been reported to exert multiple cardioprotective effects across different disease settings through diverse cellular mechanisms (Shi et al., 2021; Wei et al., 2022). While this study highlights the crucial role of Sirt1/γ-H2AX signaling, other potential pathways may also be involved and warrant further investigation.

## 5 Conclusion

Wogonin offers cardiac protective effects in PF by inhibiting DNA damage and cardiomyocyte apoptosis through activation of the Sirt1/γ-H2AX pathway. Given the limited therapeutic options for HF associated with IPF, these findings highlight the potential translational application of wogonin for treating cardiac comorbidities in the context of lung fibrosis.

## Data Availability

The raw data supporting the conclusions of this article will be made available by the authors, without undue reservation.

## Abbreviations

α-SMA, alpha smooth muscle actin; BLM, bleomycin; CVD, cardiovascular diseases; EF, ejection fraction; FS, fractional shortening; GADPH, glyceraldehyde-3-phosphate dehydrogenase; HF, heart failure; PH, pulmonary hypertension; PF, pulmonary fibrosis; Sirt1, Sirtuin silent information regulator 1; TUNEL, terminal deoxynucleotidyl transferase-mediated dUTP-biotin nick end labeling assay; Wog, wogonin; γ-H2AX, gamma H2A histone family member X.
