# Supplementary figures and images for: Cardiac protection of wogonin in mice with pulmonary fibrosis by regulating Sirt1/ γ-H2AX pathway
Source: Front Pharmacol. 2025 Apr 14;16:1551141. doi: 10.3389/fphar.2025.1551141 (PMC12034711; doi:10.3389/fphar.2025.1551141)

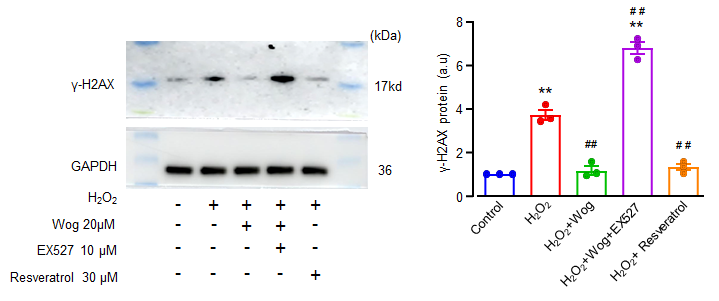

Supplement: Supplementary file 1 [file Image3.tif]

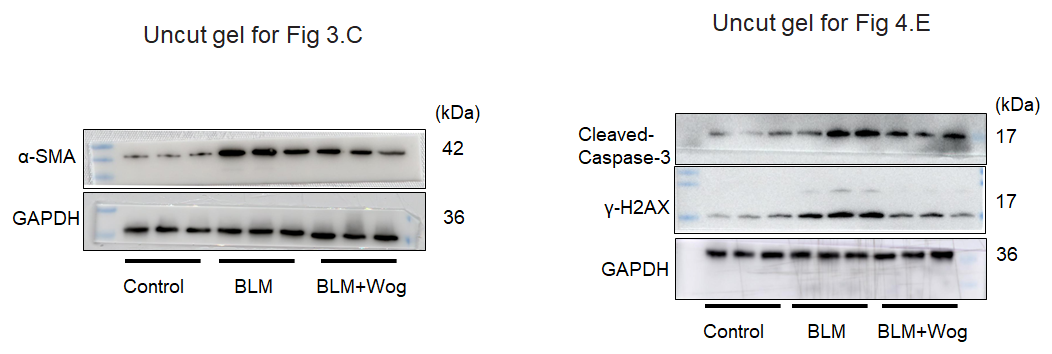

Supplement: Supplementary file 2 [file Image4.tif]

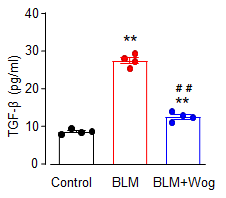

Supplement: Supplementary file 3 [file Image2.tif]

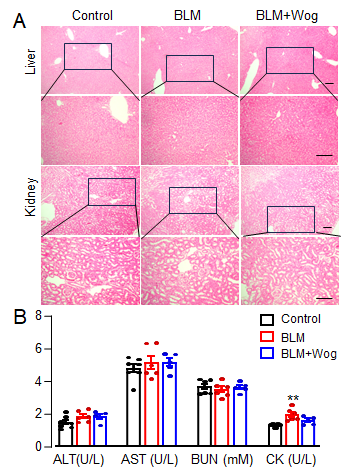

Supplement: Supplementary file 4 [file Image1.tif]

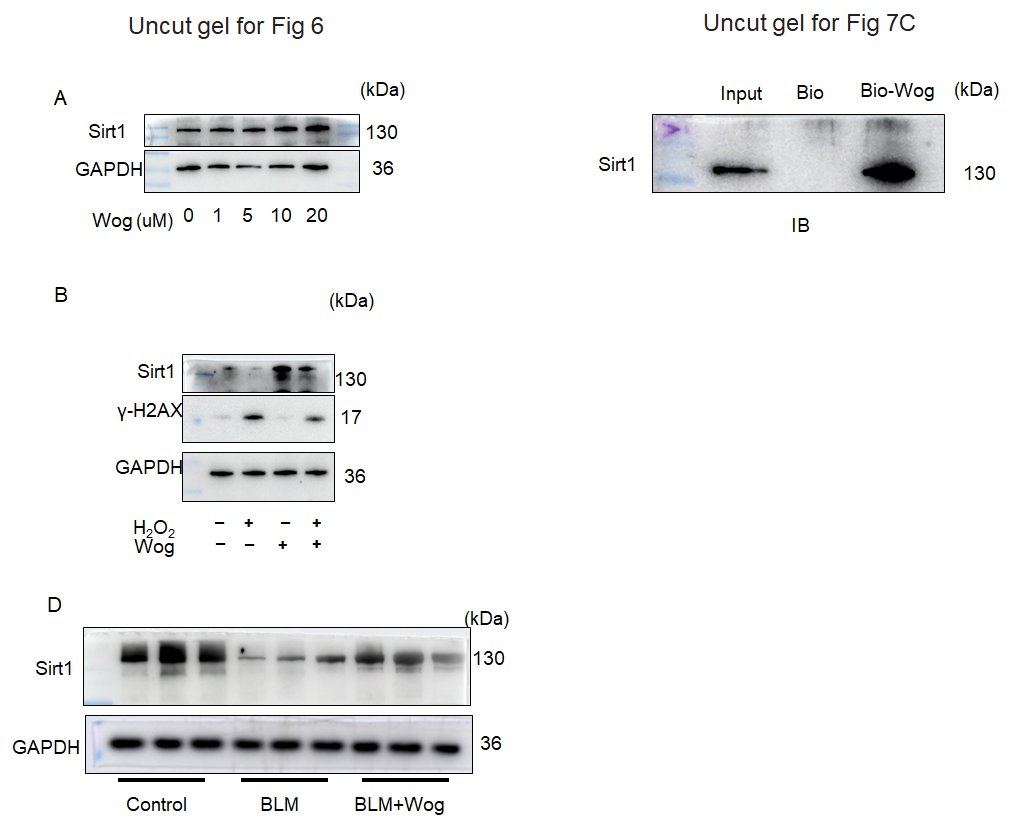

Supplement: Supplementary file 5 [file Image5.tif]
