# Supplementary material for: Cardiac protection of wogonin in mice with pulmonary fibrosis by regulating Sirt1/ γ-H2AX pathway
Source: Front Pharmacol. 2025 Apr 14;16:1551141. doi: 10.3389/fphar.2025.1551141 (PMC12034711; doi:10.3389/fphar.2025.1551141)
Supplement: Supplementary file 6 [file DataSheet1.docx]

**Supplemental Figure Legends**

**Supplemental Figure 1**. **Administration of wogonin has no toxic side effects in mice *in vivo*.** (A) Representative H&E images of the liver and kidney. scale bar, 100μm. (B) Plasma levels of alanine transaminase (ALT), aspartate transaminase (AST), blood urea nitrogen (BUN) and creatine kinase (CK) after treatment with or without bleomycin (BLM) and wogonin (Wog). n=5-7/group. ***p* < 0.01, compared with control groups, one-way ANOVA with a post-hoc Tukey’s test. All data are presented as the mean ± SEM.

**Supplemental Figure 2.** **Plasma levels of TGF-β.** n=4/group; ***p* < 0.01 compared with controls, ## *p* < 0.01, compared with BLM group, one-way ANOVA with a post-hoc Tukey’s test. All data are presented as the mean ± SEM. BLM, bleomycin; Wog, wogonin.

**Supplemental Figure 3.** **Effects of Sirt1 inhibitor or activator on DNA damage in H9C2 cells stimulated with H_2_O_2._** Western blot analysis of γ-H2AX levels in H9C2 cells induced by H_2_O_2_ following the treatment with Sirt1 specific inhibitor EX-527 (10μM) or Sirt1 activator Resveratrol (30μM) for 12 h. Mean data shown at the right. n=3/group; ***p* < 0.01 compared with controls, ## *p* < 0.01, compared with H_2_O_2_ group, one-way ANOVA with a post-hoc Tukey’s test. All data are presented as the mean ± SEM.
